# The Main Metabolites of Fluorouracil + Adriamycin + Cyclophosphamide (FAC) Are Not Major Contributors to FAC Toxicity in H9c2 Cardiac Differentiated Cells

**DOI:** 10.3390/biom9030098

**Published:** 2019-03-11

**Authors:** Ana Reis-Mendes, Félix Carvalho, Fernando Remião, Emília Sousa, Maria de Lourdes Bastos, Vera Marisa Costa

**Affiliations:** 1 UCIBIO, REQUIMTE, Laboratory of Toxicology, Department of Biological Sciences, Faculty of Pharmacy, University of Porto, 4050-313 Porto, Portugal; felixdc@ff.up.pt (F.C.); remiao@ff.up.pt (F.R.); mlbastos@ff.up.pt (M.d.L.B.); 2 Laboratory of Organic and Pharmaceutical Chemistry, Department of Chemistry, Faculty of Pharmacy, University of Porto, 4050-313 Porto, Portugal; esousa@ff.up.pt; 3 CIIMAR–Interdisciplinary Centre of Marine and Environmental Research, 4450-208 Porto, Portugal

**Keywords:** doxorubicinol, fluoro-β-alanine, acrolein, cardiotoxicity, differentiated H9c2 cells

## Abstract

In the clinical practice, the combination of 5-fluorouracil (5-FU) + Adriamycin (also known as doxorubicin, DOX) + cyclophosphamide (CYA) (known as FAC) is used to treat breast cancer. The FAC therapy, however, carries some serious risks, namely potential cardiotoxic effects, although the mechanisms are still unclear. In the present study, the role of the main metabolites regarding FAC-induced cardiotoxicity was assessed at clinical relevant concentrations. Seven-day differentiated H9c2 cells were exposed for 48 h to the main metabolites of FAC, namely the metabolite of 5-FU, α-fluoro-β-alanine (FBAL, 50 or 100 μM), of DOX, doxorubicinol (DOXOL, 0.2 or 1 μM), and of CYA, acrolein (ACRO, 1 or 10 μM), as well as to their combination. The parent drugs (5-FU 50 μM, DOX 1 μM, and CYA 50 μM) were also tested isolated or in combination with the metabolites. Putative cytotoxicity was evaluated through phase contrast microscopy, Hoechst staining, membrane mitochondrial potential, and by two cytotoxicity assays: the reduction of 3-(4,5-dimethylthiazol-2-yl)-2,5-diphenyl tetrazolium bromide (MTT) and the neutral red (NR) lysosomal incorporation. The metabolite DOXOL was more toxic than FBAL and ACRO in the MTT and NR assays. When in combination, neither FBAL nor ACRO increased DOXOL-induced cytotoxicity. No nuclear condensation was observed for any of the tested combinations; however, a significant mitochondrial potential depolarization after FBAL 100 μM + DOXOL 1 μM + ACRO 10 μM or FBAL 100 μM + DOXOL 1 μM exposure was seen at 48 h. When tested alone DOX 1 μM was more cytotoxic than all the parent drugs and metabolites in both the cytotoxicity assays performed. These results demonstrated that DOXOL was the most toxic of all the metabolites tested; nonetheless, the metabolites do not seem to be the major contributors to FAC-induced cardiotoxicity in this cardiac model.

## 1. Introduction

Chemotherapy is still the most common treatment against cancer [1]. Polychemotherapy regimens are commonly used in the clinical practice nowadays [2] and have led to higher survival and lower recurrence rates, namely in breast cancer. Breast cancer is the most common cancer and cause of cancer death worldwide in women [2]. Doxorubicin (DOX, also known as Adriamycin) is among the most active single agents used in the treatment of breast cancer [3] and it is commonly used in combination with 5-fluorouracil (5-FU) and/or cyclophosphamide (CYA) [4,5]. This combination is known as FAC regimen and it is given at 500/50/500 mg/m^2^ through the intravenous (i.v.) route, every three weeks, for six cycles [2,6]. This regimen has been used with good survival rates in breast cancer [7].

All the three drugs, 5-FU, DOX, and CYA, are reported as potentially cardiotoxic [8,9,10]. The drug 5-FU causes coronary artery spasm, autoimmune-mediated injury of the myocardium, endothelial damage, thrombogenic effects, cardionecrosis, and global systolic dysfunction [11]. The anticancer drug DOX causes acute and chronic cardiac toxicity (e.g., electrocardiographic abnormalities, arrhythmias, ventricular dysfunction, and an increase in plasma brain natriuretic peptide, cardiomyopathy, and heart failure), with DOX-induced cardiotoxicity being dose cumulative-dependent [12]. In fact, it is not recommended to surpass a 550 mg/m^2^ lifetime dose [13]. On the other hand, CYA (high doses) causes asymptomatic pericardial effusions, heart failure, and fatal myopericarditis [14]. 

In the study by Dalley et al. the authors evaluated the response and toxicity of FAC combination in 26 patients with metastatic breast carcinoma. Only one patient developed cardiotoxicity and that patient received >450 mg/m^2^ DOX cumulative dose [15]. Phase II and phase III clinical studies comparing DOX plus docetaxel (298 mg/m^2^ DOX cumulative dose) with FAC combination (299 mg/m^2^ DOX cumulative dose) as first-line chemotherapy in patients with metastatic breast cancer caused congestive heart failure in 3% patients with DOX plus docetaxel and 6% with FAC, although authors did not observe significant statistical difference between the two treatment regimens [16]. In the study of Martin et al., 1925 patients with node-positive breast cancer were recruited and randomly assigned to receive six cycles of FAC or four cycles of FAC followed by eight weekly administrations of paclitaxel. After a cumulative dose of 300 mg/m^2^ of DOX, seven deaths occurred due to cardiovascular diseases (three infarctions, two arrhythmias, one aneurism, and one cerebrovascular haemorrhage) [17]. Most authors assume that the cardiac damage seen is oxidative stress-related [18,19]. The formation of free radicals induced by DOX may cause membrane peroxidation and cardiac myocyte rupture [20]. Amin et al. observed in the plasma of patients treated with FAC an increase of nitric oxide and lipid peroxidation with a decrease of the antioxidants catalase and glutathione [19]. Although most authors assume that the total cumulative dose of DOX in human is the FAC’s major contributor to cardiotoxicity [21,22], other works showed higher incidence of cardiotoxicity in the FAC regimen when compared to DOX-induced toxicity by itself [23,24].

**Figure 1:**
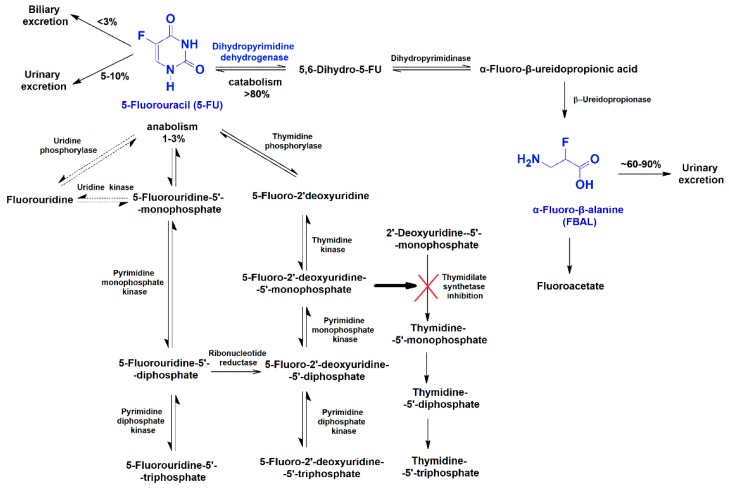
The 5-fluorouracil (5-FU) anabolism and catabolism pathways [30,31,32].

Drug metabolism may contribute to drug toxicity, as it can generate toxic metabolites [8,9]. The role of the metabolites of anticancer drugs on their induced cardiotoxicity has not been fully recognized. The cardiotoxic potential of 5-FU is known, and, 5-FU is the second most common cause of chemotherapy-induced cardiotoxicity [11]. It increases reactive oxygen species (ROS) in rat cardiomyocytes [25] and leads to diminished activity of cardiac superoxide dismutase and glutathione peroxidase in guinea pigs [26]. In animal models [27] and in humans [28,29], 5-FU also changed pro-inflammatory cytokine levels. The enzyme dihydropyrimidine dehydrogenase catabolizes 5-FU to α-fluoro-β-alanine (FBAL) (Figure 1) [30,31,32]. Plasma concentrations of 5-FU and FBAL have been determined in treated cancer patients, ranging between 22.3 and 203 μM [33] and between 3.29 and 170 μM [34,35,36], respectively (Table 1). A 70-year-old man presenting 5-FU-induced cardiotoxicity had a high level of serum FBAL. Nevertheless, after oral administration of S-1 (a derivative of 5-FU that inhibits dihydropyrimidine dehydrogenase), the serum FBAL concentration decreased and, thereafter, no other cardiac symptoms were observed [34].

**Figure 2:**
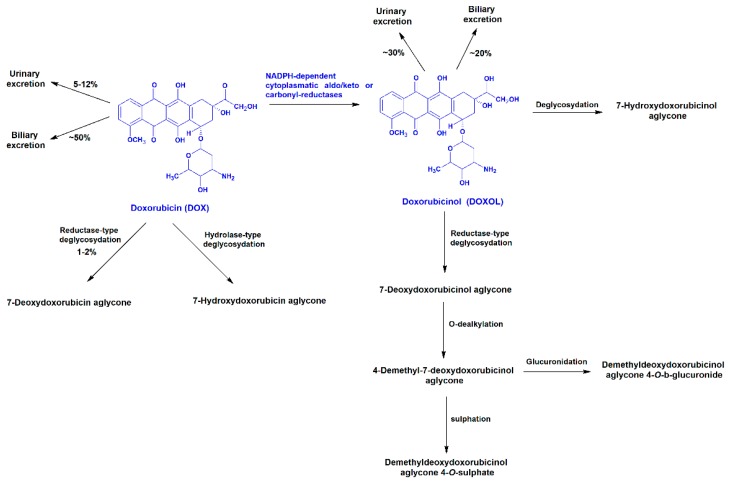
Metabolism of doxorubicin (DOX) [40,43,44].

The anticancer drug DOX has known cardiotoxic side effects [12]. The administered DOX is eliminated approximately 50% as the unchanged drug [37] and approximately 30% is excreted as a secondary alcohol metabolite doxorubicinol (DOXOL), into the urine [40]. The metabolite DOXOL is formed by the enzymatic reduction of a carbonyl group in the side chain of DOX by carbonyl reductases (Figure 2) [9], being vastly accumulated in the human heart for long periods of time, possibly due to its high hydrophilicity [41]. Plasma concentrations of DOX and its metabolite, DOXOL, have been determined, ranging between 0.24 and 1.36 μM [42] and between 0.02 and 0.1 μM [37,38], respectively (Table 1).

**Figure 3:**
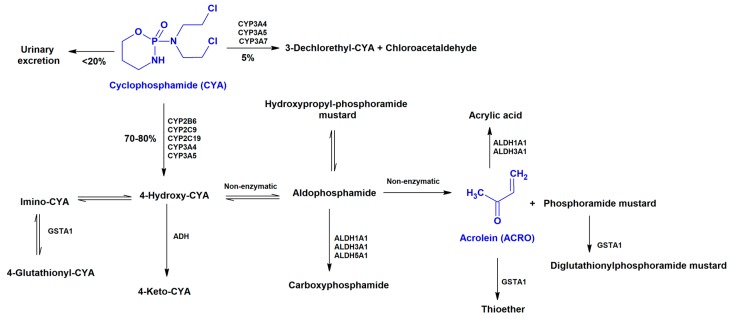
Metabolism of cyclophosphamide (CYA) [33,46,53,54]. CYP: Cytochrome P450; GST: Glutathione S-transferase; ALDH: Aldehyde dehydrogenase; ADH: Alcohol dehydrogenase.

The anticancer drug CYA is extensively metabolized to active metabolites (4-hydroxy-CYA, aldophosphamide, phosphoramide mustard, and acrolein (ACRO)) by the hepatic cytochrome P450 enzyme system, or it may be oxidized by aldehyde dehydrogenase 1 to inactive metabolites (4-ketocyclophosphamide, carboxyphosphamide) (Figure 3) [45,46]. Human plasma concentrations of CYA and its metabolite, ACRO, have been determined in treated patients and ranged between 9 and 172 μM [33,47] and between 6.2 and 10.2 μM [39], respectively (Table 1). The metabolite 4-hydroxy-CYA is the most commonly metabolite used to study the cardiotoxic effects of CYA metabolization [48,49]; however, 4-hydroxy-CYA is very unstable, and spontaneously decomposes into phosphoramide mustard and ACRO [50]. Therefore, ACRO was selected herein, since it has been described as a putative cause of cardiac toxicity of CYA [51,52].

Whether the metabolism of 5-FU, DOX or CYA contributes to the FAC-induced cytotoxicity has not yet been properly investigated, and to the best of our knowledge, no studies were done to assess the contribution of the main metabolites of DOX, 5-FU or CYA to their cardiotoxicity. Our previous study in H9c2 cells, using clinically relevant concentrations of 5-FU, DOX, and CYA and their combination, demonstrated that DOX was the most toxic drug and key to the toxicity of the combination regimen FAC, while no relevant synergistic or additive effects were observed in the combination settings performed [55]. Nevertheless, in that study the possible contribution of the metabolites towards the observed cardiotoxicity of FAC in clinical settings was not tested. As such, a new study was required to determine whether the metabolites of FAC could contribute to its induced cardiotoxicity. Therefore, we assessed the cytotoxicity of 5-FU, DOX, or CYA metabolites (FBAL, DOXOL, and ACRO), alone or in combination, in H9c2 differentiated cells to determine their potential contribution to FAC-induced cardiotoxicity. In this work, we assessed the cytotoxicity of the metabolites and their mixtures and we also used mixtures containing the metabolites and parent compounds at the same time, to mimic realistic in vivo conditions [35,37,38,56]. We aimed to observe if synergistic/ antagonistic effects occur in different combinations, as several different biological mechanisms may contribute to FAC-induced cardiotoxicity.

## 2. Materials and Methods

### 2.1. Materials

Dulbecco′s Modified Eagle Medium (DMEM) high glucose, trypsin-ethylenediaminetetraacetic acid, trypan blue solution 0.4% (w/v), sodium dodecyl sulphate, hydrochloric acid (HCl), sodium bicarbonate, 3-(4,5-dimethylthiazol-2-yl)-2,5-diphenyl tetrazolium bromide (MTT), neutral red (NR) solution, Hoescht 33258 solution, dimethyl sulfoxide, retinoic acid, paraformaldehyde, carbonyl cyanide 3-chlorophenylhydrazone (CCCP), ACRO, and FBAL were obtained from Sigma-Aldrich (Taufkirchen, Germany). 5,5′,6,6′-Tetrachloro-1,1′,3,3′-tetraethylbenzimidazolylcarbo-cyanine iodide (JC-1) was obtained from Thermo Fisher Scientific (Waltham MA, USA). Phosphate-buffered saline (PBS) without calcium and magnesium and penicillin/streptomycin were obtained from Biochrom (Berlin, Germany). Foetal bovine serum (FBS), Hanks’ balanced salt solution (HBSS) were acquired to Gibco (Paisley, UK). Doxorubicinol hydrochloride was obtained from Tebu-bio (Lisbon, Portugal). All plastic sterile material used in cell culture was obtained from Corning-Costar (Corning, NY, USA).

### 2.2. Methods

#### 2.2.1. Cell Culture Experimental Protocols

The potential cytotoxicity of FBAL (50 or 100 μM), DOXOL (0.1 or 1 μM), and ACRO (1 or 10 μM) and of their parent drugs (5-FU 50 μM, DOX 1 μM, and CYA 50 μM), and respective mixtures, was assessed in differentiated H9c2 cells. The rat cardiomyocyte derived H9c2 cell line was obtained from the European Collection of Cell Cultures of Sigma-Aldrich (Taufkirchen, Germany). The H9c2 cells were derived from the embryonic BD1X rat heart tissue obtained by Kimes and Brandt [57]. To maintain H9c2 cells in proliferative state, they were kept in complete medium (DMEM high glucose supplemented with 10% FBS and antibiotics (100 units/mL penicillin and 100 μg/mL streptomycin)) in an incubator at 37 °C with 5% CO_2_. To prevent loss of myoblast cells, all experiments were carried out before the cells reached 70–80% confluence and the cell line was used before passage 18 [58,59]. Moreover, no more than 10 passages were done after de-freezing the cells. Cells were seeded in a density of 24,000 cells/mL [59] and 24 h after, differentiation began. Cell differentiation was achieved with DMEM supplemented with 1% FBS, 10 nM retinoic acid and antibiotics, while medium was changed every other day for seven days. The differentiation was done to enhance H9c2 cardiac adult characteristics, as already described [58,60,61] making them a more reliable cardiac model. The H9c2 cells are a common model to study the cardiotoxicity of anticancer drugs [62,63,64]. Actually, during the differentiation process, cells evidenced cardiac type-specific differentiation markers such as myogenin and MyoD [60]. Differentiation decreases cell proliferation and induces several morphological and biochemical changes. The differentiation of H9c2 cells leads to increased glycolytic and mitochondrial metabolism, increases genes associated with mitochondrial energy production including respiratory chain complexes subunits, mitochondrial creatine kinase, carnitine palmitoyltransferase I and uncoupling proteins [65]. Furthermore, it leads to increased levels of transcripts and proteins involved in calcium handling, increases the expression of genes encoding for cardiac sarcomeric proteins, such as troponin T, or calcium transporters and associated machinery, including SERCA2, ryanodine receptor and phospholamban [65].

All drugs studied were dissolved in PBS. All drugs, except DOXOL were stored at −20 °C for a maximum of one month and then fresh solutions were done. DOXOL also was stored at −20 °C and thawed at a maximum of three times. The ACRO solutions were extemporaneously prepared before each experiment.

#### 2.2.2. Experimental Protocol Paradigm

After the seventh day of the differentiation protocol, H9c2 cells were incubated with the three metabolites, the metabolite of 5-FU, FBAL (50 or 100 µM); of DOX, DOXOL (0.2 or 1 µM); of CYA, ACRO (1 or 10 µM), or with the parent drugs 5-FU 50 µM, DOX 1 µM, or CYA 50 µM alone or in combination. Each compound was individually prepared and added separately to the cells, even when the combination was to be tested. The concentrations used were based on the clinical plasma levels of treated patients with those anticancer drugs (Table 1).

To determine the influence of metabolites in the toxicity of the parent drugs, since they are still present in circulation while metabolism occurs, two parent drug (5-FU, DOX, or CYA) were combined with metabolites by using two different concentrations (high or low concentrations as found in the plasma of treated patients). The parent compound was not incubated with its metabolite because we would not know the amount of metabolite that might be formed during the incubation period and therefore influence in the total outcome. Finally, the metabolites were also tested in combination. Two assays of cytotoxicity (MTT reduction and NR uptake) and morphological evaluation, using phase contrast microscopy and Hoescht nuclear staining, were performed after a 48 h incubation. Mitochondrial membrane potential was assessed by the JC-1 probe in the combination with the highest concentrations of the main metabolites of FAC; FBAL 100 µM, DOXOL 1 µM, ACRO 10 µM, and their combination. All of these determinations are described below.

#### 2.2.3. Cytotoxicity Tests

##### MTT Reduction Assay

The MTT colorimetric assay is based on the reduction of the tetrazolium salt by dehydrogenases, namely mitochondrial, with formazan formation. The cells were incubated with the parent drugs and their metabolites in mixture or alone, for 48 h, before the MTT reduction assay was performed. After the incubation period, the media was removed and 200 µL of the differentiation medium and 20 µL of MTT (5 mg/mL) were added to each well. The cells were incubated at 37 °C for 4 h, to allow the metabolism of MTT [59]. The reaction was stopped by adding 10% sodium dodecyl sulphate in 0.01 M HCl, followed by an overnight incubation at 37 °C for the dissolution of the formed formazans. The percentage of MTT reduction of control cells was set to 100% and the values were expressed as percentage of control cells.

##### Lysosomal Neutral Red Uptake Assay

The NR dye easily enters viable cell membranes and is stored in lysosomes [66]. After the 48 h drug incubation, the medium was removed and warm NR (33 µg/mL) enriched medium was then placed in each well for 3 h at 37 °C. Then, the cells were washed with warm HBSS with calcium and magnesium and the NR within the cells was released with a lysis solution [ethanol: acetic acid solution (50%:1% v/v) in water]. After a 15-min agitation, the absorbance was measured at 540 nm and 690 nm [59], in a multi-well plate reader (Biotech Synergy HT (Winooski, VT, USA)). Results were set to percentage of control cells, whose mean values were set to 100%.

#### 2.2.4. Microscopic Observation of the Cells

##### Contrast Phase Microscopy

Morphology of the cells was assessed by phase contrast microscopy to evaluate any signs of cytotoxicity after a 48 h incubation. A Nikon Eclipse TS100 equipped with a Nikon DS-Fi1 camera (Tokyo, Japan) was used.

##### Hoechst Nuclear Staining

For evaluation of changes in nuclear morphology in H9c2 differentiated cells, the Hoechst staining was used, as previously described [59,67]. Cells were then examined in a Nikon Eclipse TS100 equipped with a Nikon DS-Fi1 camera using a fluorescent filter (*λ*excitation = 346 nm and *λ*emission = 460 nm).

#### 2.2.5. Mitochondrial Membrane Potential

For the evaluation of mitochondrial membrane potential, a lipophilic cationic dye, JC-1 was used, as previously described in H9c2 differentiated cells [55]. The JC-1 dye selectively enters into mitochondria and spontaneously forms J-aggregates with intense red fluorescence in the polarized mitochondria. The JC-1 dye reversibly changes colour from red to green during mitochondrial membrane depolarization [68,69]. The protonophore CCCP was used as an uncoupler of oxidative phosphorylation in mitochondria and it was used in each condition as a positive control for mitochondrial depolarization. Two fluorescent readings were done: red was read at a λexcitation maximum = 535 nm and a λemission maximum = 595 nm and green was read at a λexcitation maximum = 485 nm and a λemission maximum = 535 nm. The fluorescence was evaluated in a multi-well plate reader (Biotech Synergy HT (Winooski, VT, USA)). The ratio of red and green fluorescence was calculated for each condition and mean control values were set to 100%.

#### 2.2.6. Statistical Analysis

The results are expressed as mean ± standard deviation (SD). The outliers were evaluated by the robust regression and outlier removal (ROUT) test. The D′Agostino and Pearson normality test was used to evaluate data distribution. When data did not follow a normal distribution, statistical analysis was performed using the Kruskal–Wallis test, followed by the Dunn′s *post hoc* test when a significant *p* was reached. On the other hand, a parametric analysis of variance (ANOVA) was performed when data distribution was normal, followed by the Tukey′s *post hoc* test. Statistical significance was reached when *p* < 0.05. All statistical analyses were performed in GraphPad Prism 7 software (San Diego, CA, USA). In the figure legends, all details of the statistical analyses can be found.

## 3. Results

### 3.1. Both α-Fluoro-β-Alanine and Doxorubicinol Were Less Cytotoxic to Differentiated H9c2 Cells Than Their Parent Drugs

The MTT reduction and the NR lysosomal uptake assays were carried out in differentiated H9c2 cells after a 48 h incubation with FBAL at two different concentrations (50 or 100 µM), DOXOL (0.2 or 1 µM), ACRO (1 or 10 µM), and the parent drugs (5-FU 50 µM, DOX 1 µM or CYA 50 µM).

**Figure 4:**
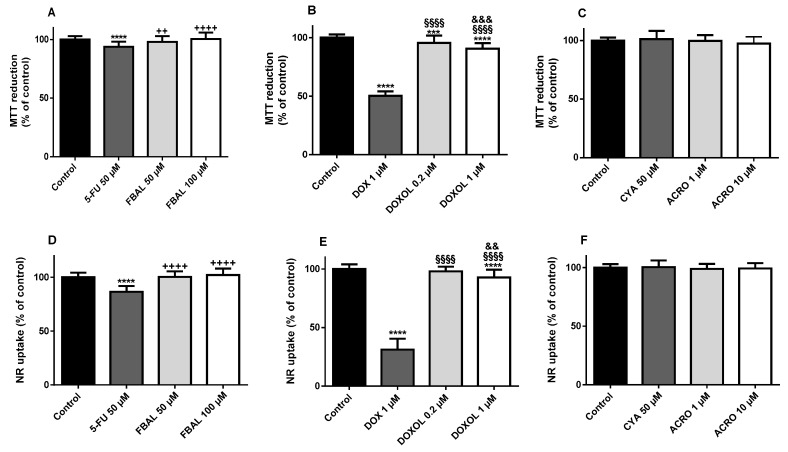
Cytotoxicity evaluated by (**A–C**) the 3-(4,5-dimethylthiazol-2-yl)-2,5-diphenyl tetrazolium bromide (MTT) reduction assay and (**D**–**F**) the neutral red (NR) uptake assay in differentiated H9c2 cells incubated for 48 h with 5-fluorouracil (5-FU) 50 μM and α-fluoro-β-alanine (FBAL) 50 μM and 100 μM (**A**,**D**); doxorubicin (DOX) 1 μM, and doxorubicinol (DOXOL) 0.2 μM and 1 μM (**B**,**E**); and cyclophosphamide (CYA) 50 μM, and acrolein (ACRO) 1 μM and 10 μM (**C**,**F**). Results are presented as mean ± SD of three to five independent experiments (total of 20–34 wells). Statistical analyses were performed using the Kruskal–Wallis test, followed by the Dunn′s post hoc test (**A**) or the analysis of variance (ANOVA) test, followed by the Tukey *post hoc* test **(B**–**F**) (*** *p* < 0.001, **** *p* < 0.0001 vs. control; ^++^ *p* < 0.01, ^++++^ *p* < 0.0001 vs. 5-FU 50 μM; ^§§§^ *p* < 0.001, ^§§§§^ *p* < 0.0001 vs. DOX 1 μM; ^&&^ *p* < 0.01, ^&&&^ *p* < 0.001 vs. DOXOL 0.2 μM).

The metabolite FBAL, at both concentrations, 50 µM (97.9 ± 5.0%) and 100 µM (100.3 ± 5.6%), did not cause significant cytotoxicity when compared to control cells (100.0 ± 2.8%) in the MTT reduction assay (Figure 4A). In the NR uptake assay, FBAL 50 µM (100.8 ± 4.6%) and 100 µM (102.1 ± 6.0%) did not cause significant cytotoxicity when compared to control (100.0 ± 4.3%) (Figure 4D). On the other hand, 5-FU 50 µM caused significant cytotoxicity, both in the MTT reduction assay (93.7 ± 4.4%) and in NR uptake assay (86.6 ± 5.4%) when compared to control (Figure 4A,D).

The anticancer drug DOX 1 µM had a significantly higher cytotoxicity when compared to its metabolite, in both the MTT reduction (50.2 ± 3.8%) or NR uptake (31.3 ± 9.3%) assays (Figure 4B,E, respectively). Moreover, DOXOL 1 µM caused statistically significant cytotoxicity, in the performed assays, after a 48 h incubation (MTT: 90.4 ± 4.8% and NR: 91.7 ± 6.2%) (Figure 4B,E).

Regarding CYA (50 µM) and its metabolite, ACRO (1 or 10 µM), they did not cause cytotoxicity neither in the MTT reduction nor in the NR assay (Figure 4C,F).

### 3.2. In Combination with 5-Fluorouracil, Cyclophosphamide or Their Metabolites, Doxorubicin Remained Key to the Cytotoxicity Observed in Differentiated H9c2 Cells

The drug DOX 1 µM was combined with 5-FU 50 µM, CYA 50 µM or their metabolites, FBAL (50 or 100 µM) or ACRO (1 or 10 µM) in differentiated H9c2 cells. In both cytotoxicity assays, all the combinations containing DOX 1 µM caused significant cytotoxicity when compared to the control cells.

**Figure 5:**
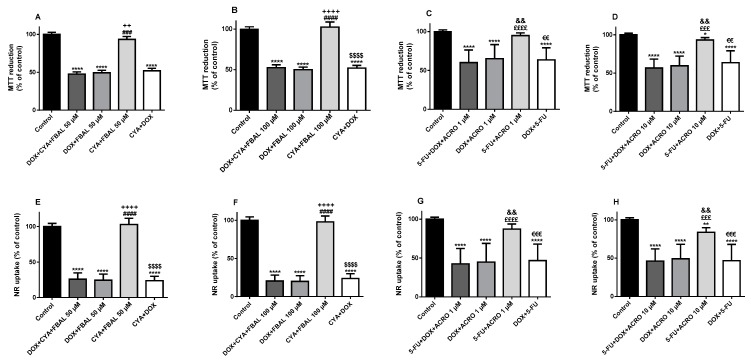
Cytotoxicity evaluated by (**A**–**D**) the MTT reduction assay (three to five independent experiments (total of 12–24 wells)) and (**E**–**H**) the NR uptake assay (six to eight independent experiments (total of 24–32 wells)) in differentiated H9c2 cells incubated for 48 h with DOX + CYA + FBAL (FBAL at concentrations of 50 or 100 μM; DOX 1 μM; and CYA 50 μM) or with 5-FU + DOX + ACRO (ACRO 1 or 10 μM; DOX 1 μM and 5-FU 50 μM). Results are presented as mean ± SD. Statistical analyses were performed using the Kruskal–Wallis test, followed by the Dunn´s post hoc test (**A,C,D,F**–**H**) or the parametric ANOVA test, followed by the Tukey’s post hoc test (**B,E**) (* *p* < 0.05, ** *p* < 0.01, **** *p* < 0.0001 vs. control; ^###^ *p* < 0.001, ^####^ *p* < 0.0001 vs. DOX + CYA + FBAL 50 and 100 μM; ^++^ *p* < 0.01, ^++++^ *p* < 0.0001 vs. DOX + FBAL 50 and 100 μM; ^$$$$^ *p* < 0.0001 vs. CYA + FBAL 50 and 100 μM; ^£££^ *p* < 0.001, ^££££^ *p* < 0.0001 vs. 5-FU + DOX + ACRO 1 and 10 μM; ^∂∂^ *p* < 0.001 vs. DOX + ACRO 1 and 10 μM; ^€€^ *p* < 0.01, ^€€€^ *p* <0.001 vs. 5-FU + ACRO 1 and 10 μM).

Either in the NR uptake or in the MTT reduction assays, the mixture DOX 1 µM + CYA 50 µM + FBAL 50 µM (MTT: 47.6 ± 2.9% and NR: 25.7 ± 8.9%) (Figure 5A,E) caused similar toxicity as compared with DOX 1 µM + CYA 50 µM + FBAL 100 µM (MTT: 49.9 ± 3.2% and NR: 20.4 ± 7.8%) (Figure 5B,F). Nevertheless, the combination DOX 1 µM + CYA 50 µM + FBAL 50 or 100 µM caused higher cytotoxicity than CYA 50 µM + FBAL 50 or 100 µM, and no significant differences were seen between any mixtures containing DOX 1 µM (Figure 5A,B,E,F).

Although ACRO 10 µM or 5-FU 50 µM did not significantly increase DOX 1 µM cytotoxicity, the combination 5-FU 50 µM + ACRO 10 µM (MTT: 95.2 ± 2.8% and NR: 83.7 ± 6.0%) caused higher cytotoxicity than the respective controls (MTT: 100.0 ± 2.1% and NR: 100.0 ± 2.5%) (Figure 5D,H) and 5-FU seems responsible for the cytotoxicity of this combination. Nevertheless, this cytotoxicity was significantly lower than that induced by 5-FU 50 µM + DOX 1 µM + ACRO 10 µM (Figure 5D,H). Moreover, the cytotoxicity of 5-FU 50 µM + DOX 1 µM + ACRO 1 µM (MTT: 60.0 ± 16.2% and NR: 42.4 ± 19.8%) was similar to that of 5-FU 50 µM + DOX 1 µM + ACRO 10 µM (MTT: 56.6 ± 11.6% and NR: 46.1 ± 16.0%) (Figure 5C,D,G–H) and DOX remained key for the cytotoxicity observed in differentiated H9c2 cells.

### 3.3. In the Combination 5-Fluorouracil + Cyclophosphamide + Doxorubicinol, 5-Fluorouracil Was Determinant to the Cytotoxicity Observed in Differentiated H9c2 Cells and Doxorubicinol Did Not Increase Its Cytotoxicity

**Figure 6:**
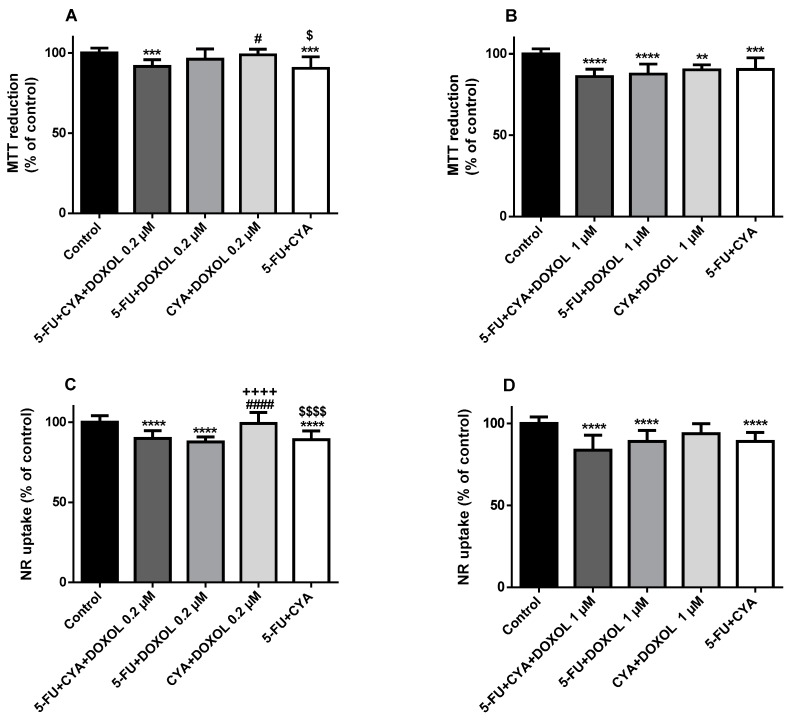
Cytotoxicity evaluated by (**A**,**B**) the MTT reduction assay and (**C**,**D**) the NR uptake assay in differentiated H9c2 cells incubated for 48 h with 5-FU + DOXOL + CYA: 5-FU at 50 μM, DOXOL (0.2 or 1 μM) and CYA at 50 μM. Results are presented as mean ± SD of three to four independent experiments (total of 10–20 wells). Statistical analyses were performed using the Kruskal–Wallis test, followed by the Dunn´s post hoc test (**A,B,D**) or the ANOVA test, followed by the Tukey’s post hoc test (**C**) (** *p* < 0.01, *** *p* < 0.001, **** *p* < 0.0001 vs. control; ^#^ *p* < 0.05, ^####^ *p* < 0.0001 vs. 5-FU + CYA + DOXOL 0.2 μM; ^++++^ *p* < 0.0001 vs. 5-FU + DOXOL 0.2 μM; ^$^ *p* < 0.05, ^$$$$^ *p* < 0.0001 vs. CYA + DOXOL 0.2 μM).

The MTT reduction and the lysosomal uptake of NR assays were done at 48 h using 5-FU 50 µM, CYA 50 µM or their combination (5-FU+CYA) with two different concentrations of DOXOL (0.2 or 1 µM) in differentiated H9c2 cells. The mixture of 5-FU 50 µM + CYA 50 µM + DOXOL 1 µM caused similar cytotoxicity as compared with 5-FU 50 µM + CYA 50 µM + DOXOL 0.2 µM (MTT: 91.6 ± 4.2% and NR: 89.9 ± 4.2%), after a 48 h exposure (Figure 6A–D). Both in the MTT reduction and the NR uptake assays, the mixture 5-FU 50 µM + CYA 50 µM + DOXOL 1 µM (MTT: 84.9 ± 2.8% and NR: 86.8 ± 4.0%) caused significant cytotoxicity when compared to the control (MTT: 100.0 ± 3.0% and NR: 100.0 ± 4.0%) (Figure 6B,D). In the MTT reduction and NR assays, 5-FU 50 µM + DOXOL 1 µM (MTT: 87.5 ± 6.1% and NR: 89.1 ± 6.7%), CYA 50 µM + DOXOL 1 µM (MTT: 90.1 ± 3.0% and NR: 93.8 ± 6.2%), and 5-FU 50 µM + CYA 50 µM (MTT: 90.4 ± 7.1% and NR: 89.1 ± 5.4%) caused statistically significant cytotoxicity when compared to the control (Figure 6B,D). Moreover, 5-FU 50 µM + CYA 50 µM caused significant cytotoxicity when compared to controls in both cytotoxicity assays (Figure 6A–D). The mixture 5-FU 50 µM + CYA 50 µM + DOXOL 0.2 µM (MTT: 91.6 ± 4.2% and NR: 89. 9 ± 4.2%) caused higher significant cytotoxicity when compared to CYA 50 µM + DOXOL 0.2 µM (MTT: 98.8 ± 3.5% and NR: 99.2 ± 6.8%), in both performed tests (Figure 6A,C) and 5-FU seemed the responsible for the cytotoxicity observed when used in combination with DOXOL (or even CYA).

### 3.4. All Combinations of α-Fluoro-β-Alanine, Doxorubicinol and Acrolein and All Mixtures Containing Doxorubicinol at the Highest Concentration Caused Cytotoxicity in Differentiated H9c2 Cells

**Figure 7:**
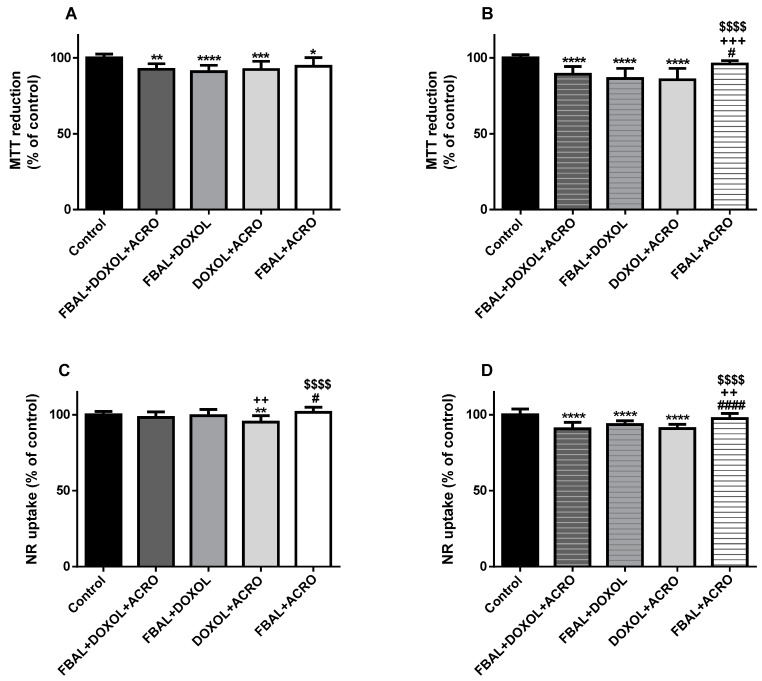
Cytotoxicity evaluated by (**A**,**B**) the MTT reduction assay and the (**C**,**D**) NR uptake assay in differentiated H9c2 cells incubated with the combination of metabolites (DOXOL + FBAL + ACRO) for 48 h. (**A**,**C**): FBAL 50 μM + DOXOL 0.2 μM + ACRO 1 μM. (**B**,**D**): FBAL 100 μM + DOXOL 1 μM + ACRO 10 μM. Results are presented as mean ± SD of three to four independent experiments (total of 12–18 wells). Statistical analyses were performed using the ANOVA test, followed by the Tukey’s post hoc test (* *p* < 0.05, ** *p* < 0.01, *** *p* < 0.001, **** *p* < 0.0001 vs. control; ^#^ *p* < 0.05, ^####^ *p* < 0.0001 vs. DOXOL + FBAL + ACRO; ^++^ *p* < 0.01, ^+++^ *p* < 0.001 vs. DOXOL + FBAL; ^$$$$^ *p* < 0.0001 vs. DOXOL + ACRO).

In the MTT reduction test, the mixture of low concentrations of FBAL 50 µM + DOXOL 0.2 µM + ACRO 1 µM (92.5 ± 3.6%), FBAL 50 µM + DOXOL 0.2 µM (91 ± 4.2%), DOXOL 0.2 µM + ACRO 1 µM (92.4 ± 5.4%), and FBAL 50 µM + ACRO 1 µM (94.4 ± 5.8%) caused cytotoxicity when compared to control cells (100 ± 2.5%) (Figure 7A). In the NR uptake assay, the mixture of FBAL 50 µM + DOXOL 0.2 µM + ACRO 1 µM (98.3 ± 3.6%) did not cause significant cytotoxicity when compared to control (100 ± 2.2%) (Figure 7C).

In both cytotoxicity assays, the mixture of high concentrations of FBAL 100 µM + DOXOL 1 µM + ACRO 10 µM (MTT: 89.3 ± 5.1% and NR: 90.7 ± 4.2%), FBAL 100 µM + DOXOL 1 µM (MTT: 86.4 ± 6.7% and NR: 93.5 ± 2.6%), and DOXOL 1 µM + ACRO 10 µM (MTT: 85.6 ± 7.5% and NR: 90.9 ± 2.8%) caused cytotoxicity when compared to control (MTT: 100 ± 2.1% and NR: 100 ± 3.8%) (Figure 7B,D). In addition, the mixture of high concentrations of the metabolites, FBAL 100 µM + DOXOL 1 µM + ACRO 10 µM (90.7 ± 4.2%), produced higher cytotoxicity when compared to FBAL 100 µM + ACRO 10 µM (96 ± 2.2%) (Figure 7B,D).

**Figure 8:**
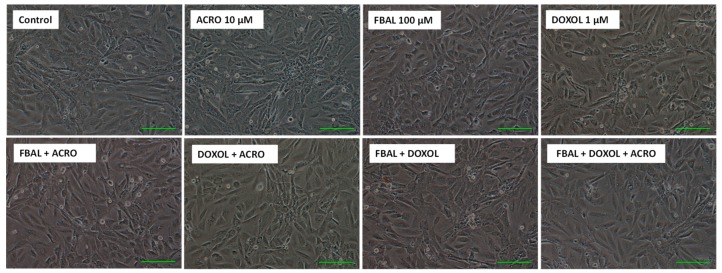
Phase contrast microscopy of differentiated H9c2 cells after a 48 h exposure to metabolites (FBAL 100 μM, DOXOL 1 μM, ACRO 10 μM) or in combination. Images are representative of two independent experiments. Scale bar: 100 μM.

**Figure 9:**
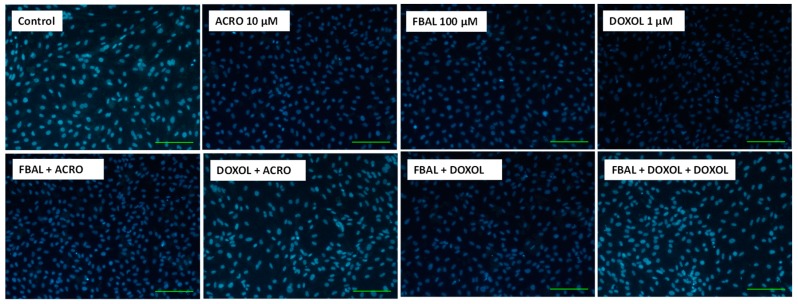
Fluorescence microscopy (Hoechst 33258 staining) of differentiated H9c2 cells after a 48 h exposure to the metabolites (FBAL 100 μM, DOXOL 1 μM, ACRO 10 μM) and their combination. Images are representative of two independent experiments. Scale bar: 100 μM.

Phase contrast microscopy after a 48 h exposure to the high concentrations of the metabolites alone or in mixture did not reveal any signs of cytotoxicity when compared to control cells (Figure 8), and differentiated H9c2 cells maintained their large rounded shape. Moreover, according to the Hoescht staining, FBAL + DOXOL + ACRO did not cause any significant nuclear morphology change or decrease in the number of cells when compared to control cells (Figure 9).

### 3.5. The Mixture of the Metabolites of 5-Fluorouracil, Doxorubicin and Cyclophosphamide (α-Fluoro-β-Alanine 100 µM + Doxorubicinol 1 µM + Acrolein 10 µM) Caused a Significant Decrease in Mitochondria Potential in Differentiated H9c2 Cells

**Figure 10:**
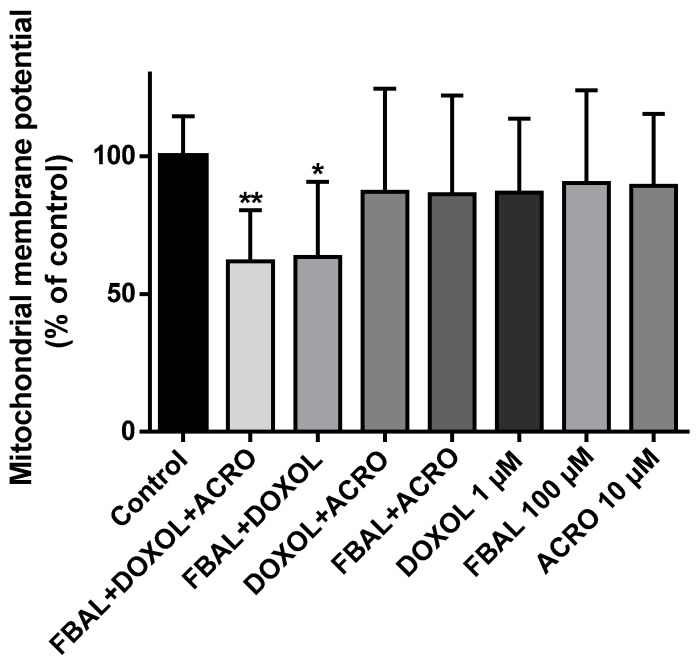
Mitochondrial membrane potential evaluation using the JC-1 dye in differentiated H9c2 cells exposed to metabolites (FBAL 100 μM, DOXOL 1 μM, ACRO 10 μM) or their combination for 48 h. Results are presented as mean ± SD of five independent experiments (total of 14–15 wells). Statistical analyses were performed using the Kruskal–Wallis test, followed by the Dunn´s post hoc test (* *p* < 0.05, ** *p* < 0.01 vs. control).

The mitochondrial membrane potential was assessed at 48 h using the highest concentrations of metabolites by themselves (FBAL 100 µM, DOXOL 1 µM, ACRO 10 µM), as well as their combination. A significant decrease in mitochondrial potential in the combinations FBAL 100 µM + DOXOL 1 µM + ACRO 10 µM (61.7 ± 18.6%) and FBAL100 µM + DOXOL 1 µM (63.4 ± 27.3%) when compared to control (100.0 ± 14.4%) was seen (Figure 10). The combination of FBAL and DOXOL seems to be responsible for that mitochondrial membrane depolarization.

## 4. Discussion

To the best of our knowledge, this work was the first to assess the contribution of the metabolites (FBAL, DOXOL, ACRO) to FAC-elicited cardiotoxicity in differentiated H9c2 cardiac cells. The major findings of this work were: (1) DOXOL was more toxic than FBAL and ACRO in both cytotoxicity assays (MTT reduction and NR uptake) and concentrations tested; (2) 5-FU and DOX were more cytotoxic than their correspondent metabolites; (3) in combination with 5-FU, CYA, or their metabolites, DOX was primarily responsible for the cytotoxicity observed in differentiated H9c2 cells; (4) for either the lowest or the highest concentrations, the metabolite mixture caused similar toxicity when compared to all combinations with DOXOL or even DOXOL alone; and (5) a significant decrease in mitochondria potential was seen after incubation with FBAL 100 µM + DOXOL 1 µM + ACRO 10 µM and FBAL 100 µM + DOXOL 1 µM treatment while no signs of nuclear condensation were observed at any of the tested combinations. In order to evaluate the toxic effects of FBAL, DOXOL, ACRO in differentiated H9c2 cells, the MTT reduction and NR incorporation tests were performed and compared to the parent drugs at clinically relevant concentrations. The anticancer drug 5-FU 50 µM caused a mild but significant cytotoxicity, while its metabolite, FBAL, (50 or 100 µM) caused no toxicity. Nonetheless, the pathogenesis of cardiotoxicity caused by 5-FU is not clear. Actually, unchanged 5-FU elimination only represents 5–10% of the administered dose [32] and the inhibition of dihydropyrimidine dehydrogenase has been reported to be protective against the 5-FU-inflicted cardiotoxicity in humans [34,70] and in animal models [71]. Another in vitro study using keratocytes with high thymidine phosphorylase activity also showed higher 5-FU toxicity when compared to FBAL [72]. Nonetheless, our in vitro data does not corroborate the higher cytotoxicity of the metabolite when compared to 5-FU.

We observed that DOXOL was more cytotoxic than the other metabolites tested, but less toxic than DOX. These results were corroborated by a previous in vitro study [73]. Bains et al. exposed eight human cell lines derived from different tissues and one rat embryonic cardiac cell line (undifferentiated H9c2 cells) to DOX at several concentrations up to 150 μM and to DOXOL at several concentrations up to 3000 μM, for 48 h. In that study, DOX showed to be significantly more toxic to all tested cell lines than its metabolite, DOXOL, according to the MTT reduction assay [73]. Cytosolic fractions of ex vivo human myocardial samples obtained during surgery for coronary bypass grafting were reconstituted with reduced β-nicotinamide adenine dinucleotide phosphate (NADPH). DOX (25 µM) was incubated for 4 h and DOXOL was found within those fractions (0.09 µM) showing that DOX metabolism can occur in loco in the human heart [74]. Moreover, DOXOL causes iron pathway dysregulation in cytosolic fractions from human myocardium [75,76] that may contribute to oxidative stress and cardiotoxicity [10]. In fact, in vivo studies seem to determine the putative role of DOXOL in DOX-induced cardiotoxicity. Olson et al. gave intraperitoneal DOX 20 mg/kg to transgenic mice with only one functional copy of carbonyl reductase. These mice had decreased circulating levels of DOXOL and were protected from gross and cellular heart damage caused by DOX [77]. Similarly, the administration of DOX (15 mg/kg i.v.) to transgenic animals overexpressing heart-specific human carbonyl reductase led to an increase conversion of DOX to DOXOL and higher development of cardiomyopathy [78]. Although the toxicity mechanisms were not determined, these two studies showed that DOXOL may play a major role in cardiotoxicity of DOX [77,78], perhaps by intensifying the damage caused by ROS. Moreover, the canonical theory for the cardiac toxicity of DOX has been attributed to its ability to generate ROS and promote iron-catalysed oxidative damage and DOXOL was found to alter the function of cytoplasmic iron regulatory proteins [79], and promote oxidative stress. Actually, DOXOL interacts with *cis*-aconitase-iron regulatory protein-1 and irreversibly inactivates aconitase activity causing the delocalization of iron from the active centre of aconitase with reoxidation of DOXOL to DOX in cytosolic fractions from human myocardium [75,76]. The inactivation of cis-aconitase-iron regulatory protein-1 leads to metabolic disruption, loss of iron homeostasis, and impairment of the contraction-relaxation cycle of the heart by misplacement of iron [13]. The excess of intracellular iron leads to the formation of ROS and tissue damage mediated by oxidative stress [80]. Nevertheless, in the in vitro study herein presented, DOX showed to be more toxic than its metabolite DOXOL. The hydrophilic nature of DOXOL can lead to its intracellular accumulation, when formed within the cell [41]; however, DOX enters more easily in the cells and, therefore, possibly caused more cytotoxicity in our model. Still, DOXOL was the most toxic of the FAC-metabolites tested, even when used in lower concentrations. As referred above, carbonyl reductase is an important enzyme in the catalytic reaction of DOX metabolism. In a work by Zhou and colleagues, they found that carbonyl reductase 1 mediated the metabolism of DOX in non-differentiated H9c2 cells and that the inhibition of that enzyme through siRNA robustly decreased DOX-induced cytotoxicity [81]. As a consequence, it may be expected that, in our cell model, the toxicity observed after DOX may also involve its metabolite, DOXOL.

The drug ACRO is a ubiquitous environmental pollutant that has been implicated in increased cardiovascular disease in humans [52]. It causes cardiotoxicity, both in vivo [52] as in vitro [48]. In the H9c2 cardiac cell line, CYA metabolites, ACRO and 4-hydroxy-CYA, have been implicated in the cytotoxicity of CYA [49,56]. In our study, CYA and its metabolite ACRO did not cause cytotoxicity according to the MTT and NR assays. Kurauchi et al. exposed undifferentiated H9c2 cells to three CYA metabolites: carboxyethylphosphoramide, 4-hydroxy-CYA and ACRO for 24 and 48 h. Only 4-hydroxy-CYA at 10 and 30 μM, and ACRO at 30 μM were cytotoxic at 24 h and 48 h. The authors also observed that there was a conversion of 4-hydroxy-CYA to ACRO in culture medium, attributing the higher cytotoxicity of 4-hydroxy-CYA, at least in part, to its conversion to ACRO [49]. Nishikawa et al. [56] incubated undifferentiated H9c2 cells with 10, 30, or 100 μM of ACRO and 125, 250, or 500 μM of CYA for 24 or 48 h and toxicity was evaluated by the MTT reduction test. ACRO caused cytotoxicity at 30 μM and 100 μM, while the parent compound did not elicit cytotoxicity at any of the tested concentrations [56]. Also, Wang et al. exposed mouse cardiomyocytes to ACRO at concentrations ranged between 1 and 100 μM and showed that this CYA metabolite causes a concentration-dependent increase of ROS production, calcium loading, and apoptosis [51]. ACRO seems to contribute to cardiac toxicity, possibly through oxidative stress mechanisms, with decrease levels of the antioxidant glutathione and increase ROS generation [49,56]. In fact, ACRO toxicity may be mediated by redox-sensitive transcription factors such as nuclear factor-kappa B (NF-κB) or activator protein-1 (AP-1), via generation of ROS and nitric oxide. In inflammatory cells isolated from lungs treated with ACRO, it increased ROS formation via induction of NF-κB signalling, and increased expression of inflammatory cytokines [82]. On the other hand, sublethal concentrations of ACRO caused a biphasic activation on NF-κB activation in A549 human lung adenocarcinoma cells, NF-κB decreasing dramatically at 1–2 h post-treatment and returning to near normal binding by 8–12 h [83]. In that same cellular model, AP-1 activity was decreased [84]. ACRO can also be an initiator of oxidative stress by forming adducts with cellular nucleophilic groups due to its high reactivity [84,85]. When ACRO was administered orally (1 mg/kg daily for 48 days) to C57BL/6 mice, myocardial oxidative stress, nitrative stress, and increase of plasma and myocardial protein–acrolein adduct formation were present 1 and 24 h after exposure, thus demonstrating that ACRO translocation to heart occurs via distribution and toxic effects may result [52]. Nevertheless, in our work, ACRO did not cause cytotoxicity in differentiated H9c2 cells at the concentrations tested.

The anticancer drug DOX is a known mitochondrial toxicant [86] and it caused depolarization of the mitochondrial membrane, determinant for FAC mitochondrial toxicity [55]. Herein, we evaluated the mitochondrial membrane potential of H9c2 cells after exposure to the FAC metabolites, tested at clinically relevant concentrations. Through JC-1 staining, a significant decrease in mitochondrial membrane potential after incubation with FBAL 100 µM + DOXOL 1 µM + ACRO 10 µM and FBAL 100 µM + DOXOL 1 µM occurred. A synergistic effect of FBAL and DOXOL regarding cardiomyocyte mitochondrial membrane depolarization was observed. Although the knowledge of the DOXOL cardiotoxic mechanisms are at present very limited, it is reasonable to assume that DOXOL may increase ROS, which may contribute to the genesis of mitochondrial membrane depolarization. Redox homeostasis is a critical factor for normal functioning of mitochondria [87]. Nonetheless, other mechanisms may be involved in DOXOL injury to the mitochondria. The metabolite DOXOL has shown to interact with NADH dehydrogenase of heart mitochondria and sarcosome-containing NADPH cytochrome P450 reductase, resulting in superoxide anion production, through one-electron redox cycling of the quinone moiety [88]. Moreover, there is evidence that DOXOL completely inhibits the cardiac mitochondrial Mg^2+^-ATPase activity referable to the FoF1 reversible proton pump, which is responsible for ATP synthesis via oxidative phosphorylation [89,90], thus leading to disruption in energetic metabolism. To the best of our knowledge, there is no data regarding FBAL-induced mitochondrial toxicity, although in vivo 5-FU caused damage to the mitochondria of the heart [91]. Further investigations are needed to completely clarify the pathophysiological role of DOXOL or FBAL-induced mitochondrial toxicity, as it has been a scarcely studied subject.

In conclusion, this work showed that the DOX metabolite, DOXOL, is the most toxic metabolite tested when compared to the other two metabolites (FBAL and ACRO) of the FAC combination. Still, DOXOL was less cytotoxic than DOX and our results demonstrate that the metabolites are not the major contributors to FAC-induced cardiotoxicity in differentiated H9c2 cells.

## Figures and Tables

**Table 1:** Plasma concentrations of α-fluoro-β-alanine (FBAL), doxorubicinol (DOXOL), or acrolein (ACRO) in chemotherapy-treated patients.

Drug	Patients and Dose	Plasma Concentrations	Reference
FBAL	One patient received a continuous intravenous (i.v.) infusion of 5-FU (1000 mg/day)	3.29–18.26 μM	[34]
	Four patients received 5-FU once daily for 5 days (one cycle) at 8–13 mg/kg, repeated every three weeks (group A) One patient received 5-FU once weekly at 14 mg/kg (group B) Four patients received 20–26 mg 5-FU/kg once every three weeks (group C) Twelve patients received a pre-treatment of 5.1–12.5 mg mitoxantrone/kg followed by 15–30 mg FU/kg, repeated once every three weeks (group D)	19–170 μM	[35]
	Ten patients received protracted venous infusions of 5-FU at a dose of 250 mg/m^2^/day for 5 days	8.01 ± 0.12 μM–10.81 ± 3.27 μM	[36]
DOXOL	Seven patients received DOX 20 mg/m^2^, bleomycin 15 U/m^2^ and vincristine 2 mg as a 30-min i.v. infusion each	0.056 ± 0.005 μM	[37]
	Sixty-five patients received DOX 60 mg/m^2^ over 15 min followed by CYA 600 mg/m^2^ over 15 min	0.1 μM	[38]
ACRO	Five patients received CYA by i.v. infusion (60 mg/kg over 1 h for two consecutive days)	6.2–10.2 μM	[39]

## References

[1] Shih Y.C., Smieliauskas F., Geynisman D.M., Kelly R.J., Smith T.J. (2015). Trends in the cost and use of targeted cancer therapies for the privately insured nonelderly: 2001 to 2011. J. Clin. Oncol..

[2] Anampa J., Makower D., Sparano J.A. (2015). Progress in adjuvant chemotherapy for breast cancer: An overview. BMC Med..

[3] Chao T.C., Wang W.S., Yen C.C., Chiou T.J., Liu J.H., Chen P.M. (2003). A dose-escalating pilot study of sterically stabilized, pegylated liposomal doxorubicin (Lipo-Dox) in patients with metastatic breast cancer. Cancer Investig..

[4] Tampaki E.C., Tampakis A., Alifieris C.E., Krikelis D., Pazaiti A., Kontos M., Trafalis D.T. (2018). Efficacy and safety of neoadjuvant treatment with bevacizumab, liposomal doxorubicin, cyclophosphamide and paclitaxel combination in locally/regionally advanced, HER2-negative, grade III at premenopausal status breast cancer: A phase II study. Clin. Drug Investig..

[5] Veronese P., Hachul D.T., Scanavacca M.I., Hajjar L.A., Wu T.C., Sacilotto L., Veronese C., Darrieux F. (2018). Effects of anthracycline, cyclophosphamide and taxane chemotherapy on QTc measurements in patients with breast cancer. PLoS ONE.

[6] Martin M., Villar A., Sole-Calvo A., Gonzalez R., Massuti B., Lizon J., Camps C., Carrato A., Casado A., Candel M.T. (2003). Doxorubicin in combination with fluorouracil and cyclophosphamide (i.v. FAC regimen, day 1, 21) versus methotrexate in combination with fluorouracil and cyclophosphamide (i.v. CMF regimen, day 1, 21) as adjuvant chemotherapy for operable breast cancer: A study by the GEICAM group. Ann. Oncol..

[7] Liutkauskiene S., Grizas S., Jureniene K., Suipyte J., Statnickaite A., Juozaityte E. (2018). Retrospective analysis of the impact of anthracycline dose reduction and chemotherapy delays on the outcomes of early breast cancer molecular subtypes. BMC Cancer.

[8] Hrynchak I., Sousa E., Pinto M., Costa V.M. (2017). The importance of drug metabolites synthesis: The case-study of cardiotoxic anticancer drugs. Drug Metab. Rev..

[9] Reis-Mendes A.F., Sousa E., de Lourdes Bastos M., Costa V.M. (2015). The role of the metabolism of anticancer drugs in their induced-cardiotoxicity. Curr. Drug Metab..

[10] Costa V.M., Carvalho F., Duarte J.A., Bastos M.L., Remiao F. (2013). The heart as a target for xenobiotic toxicity: The cardiac susceptibility to oxidative stress. Chem. Res. Toxicol..

[11] Sorrentino M.F., Kim J., Foderaro A.E., Truesdell A.G. (2012). 5-Fluorouracil induced cardiotoxicity: Review of the literature. Cardiol. J..

[12] Tahover E., Patil Y.P., Gabizon A.A. (2015). Emerging delivery systems to reduce doxorubicin cardiotoxicity and improve therapeutic index: Focus on liposomes. Anticancer Drugs.

[13] Minotti G., Menna P., Salvatorelli E., Cairo G., Gianni L. (2004). Anthracyclines: Molecular advances and pharmacologic developments in antitumor activity and cardiotoxicity. Pharmacol. Rev..

[14] Wadia S. (2015). Acute cyclophosphamide hemorrhagic myopericarditis: Dilemma case report, literature review and proposed diagnostic criteria. J. Clin. Diagn. Res..

[15] Dalley D.N., Levi J.A., Aroney R.S. (1980). Combination chemotherapy with cyclophosphamide, adriamycin, and 5-fluorouracil (CAF) in advanced breast carcinoma. Med. J. Aust..

[16] Bontenbal M., Creemers G.J., Braun H.J., de Boer A.C., Janssen J.T., Leys R.B., Ruit J.B., Goey S.H., van der Velden P.C., Kerkhofs L.G. (2005). Phase II to III study comparing doxorubicin and docetaxel with fluorouracil, doxorubicin, and cyclophosphamide as first-line chemotherapy in patients with metastatic breast cancer: Results of a Dutch community setting trial for the clinical trial group of the comprehensive cancer centre. J. Clin. Oncol..

[17] Martin M., Ruiz A., Ruiz Borrego M., Barnadas A., Gonzalez S., Calvo L., Margeli Vila M., Anton A., Rodriguez-Lescure A., Segui-Palmer M.A. (2013). Fluorouracil, doxorubicin, and cyclophosphamide (FAC) versus FAC followed by weekly paclitaxel as adjuvant therapy for high-risk, node-negative breast cancer: Results from the GEICAM/2003-02 study. J. Clin. Oncol..

[18] Kaithwas G., Dubey K., Pillai K.K. (2011). Effect of aloe vera (*Aloe barbadensis* Miller) gel on doxorubicin-induced myocardial oxidative stress and calcium overload in albino rats. Indian J. Exp. Biol..

[19] Amin K.A., Mohamed B.M., El-Wakil M.A., Ibrahem S.O. (2012). Impact of breast cancer and combination chemotherapy on oxidative stress, hepatic and cardiac markers. J. Breast Cancer.

[20] Koti B.C., Vishwanathswamy A.H., Wagawade J., Thippeswamy A.H. (2009). Cardioprotective effect of lipistat against doxorubicin induced myocardial toxicity in albino rats. Indian J. Exp. Biol..

[21] Kolaric K., Bradamante V., Cervek J., Cieslinska A., Cisarz-Filipcak E., Denisov L.E., Donat D., Drosik K., Gershanovic M., Hudziec P. (1995). A phase II trial of cardioprotection with cardioxane (ICRF-187) in patients with advanced breast cancer receiving 5-fluorouracil, doxorubicin and cyclophosphamide. Oncology.

[22] Buzdar A.U., Kau S.W., Smith T.L., Hortobagyi G.N. (1989). Ten-year results of FAC adjuvant chemotherapy trial in breast cancer. Am. J. Clin. Oncol..

[23] Bustova I. (2009). Risk of cardiotoxicity of combination treatment radiotherapy and chemotherapy of locally advanced breast carcinoma stage III. Klin. Onkol..

[24] Mackey J.R., Martin M., Pienkowski T., Rolski J., Guastalla J.P., Sami A., Glaspy J., Juhos E., Wardley A., Fornander T. (2013). Adjuvant docetaxel, doxorubicin, and cyclophosphamide in node-positive breast cancer: 10-Year follow-up of the phase 3 randomised BCIRG 001 trial. Lancet Oncol..

[25] Lamberti M., Porto S., Marra M., Zappavigna S., Grimaldi A., Feola D., Pesce D., Naviglio S., Spina A., Sannolo N. (2012). 5-Fluorouracil induces apoptosis in rat cardiocytes through intracellular oxidative stress. J. Exp. Clin. Cancer Res..

[26] Durak I., Karaayvaz M., Kavutcu M., Cimen M.Y., Kacmaz M., Buyukkocak S., Ozturk H.S. (2000). Reduced antioxidant defense capacity in myocardial tissue from guinea pigs treated with 5-fluorouracil. J. Toxicol. Environ. Health A.

[27] Logan R.M., Stringer A.M., Bowen J.M., Gibson R.J., Sonis S.T., Keefe D.M. (2008). Serum levels of NFκB and pro-inflammatory cytokines following administration of mucotoxic drugs. Cancer Biol. Ther..

[28] Reers S., Pfannerstill A.C., Rades D., Maushagen R., Andratschke M., Pries R., Wollenberg B. (2013). Cytokine changes in response to radio-/chemotherapeutic treatment in head and neck cancer. Anticancer Res..

[29] Raghu Nadhanan R., Abimosleh S.M., Su Y.W., Scherer M.A., Howarth G.S., Xian C.J. (2012). Dietary emu oil supplementation suppresses 5-fluorouracil chemotherapy-induced inflammation, osteoclast formation, and bone loss. Am. J. Physiol. Endocrinol. Metab..

[30] Malet-Martino M., Martino R. (2002). Clinical studies of three oral prodrugs of 5-fluorouracil (capecitabine, UFT, S-1): A review. Oncologist.

[31] Miura K., Kinouchi M., Ishida K., Fujibuchi W., Naitoh T., Ogawa H., Ando T., Yazaki N., Watanabe K., Haneda S. (2010). 5-FU metabolism in cancer and orally-administrable 5-FU drugs. Cancers.

[32] Nies A.T., Magdy T., Schwab M., Zanger U.M. (2015). Role of ABC transporters in fluoropyrimidine-based chemotherapy response. Adv. Cancer Res..

[33] Jamieson D., Lee J., Cresti N., Jackson R., Griffin M., Sludden J., Verrill M., Boddy A.V. (2014). Pharmacogenetics of adjuvant breast cancer treatment with cyclophosphamide, epirubicin and 5-fluorouracil. Cancer Chemother. Pharmacol..

[34] Muneoka K., Shirai Y., Yokoyama N., Wakai T., Hatakeyama K. (2005). 5-Fluorouracil cardiotoxicity induced by α-fluoro-β-alanine. Int. J. Clin. Oncol..

[35] Hull W.E., Port R.E., Herrmann R., Britsch B., Kunz W. (1988). Metabolites of 5-fluorouracil in plasma and urine, as monitored by ^19^F nuclear magnetic resonance spectroscopy, for patients receiving chemotherapy with or without methotrexate pretreatment. Cancer Res..

[36] Yamada Y., Hamaguchi T., Goto M., Muro K., Matsumura Y., Shimada Y., Shirao K., Nagayama S. (2003). Plasma concentrations of 5-fluorouracil and F-β-alanine following oral administration of S-1, a dihydropyrimidine dehydrogenase inhibitory fluoropyrimidine, as compared with protracted venous infusion of 5-fluorouracil. Br. J. Cancer.

[37] Joerger M., Huitema A.D., Meenhorst P.L., Schellens J.H., Beijnen J.H. (2005). Pharmacokinetics of low-dose doxorubicin and metabolites in patients with AIDS-related Kaposi sarcoma. Cancer Chemother. Pharmacol..

[38] Joerger M., Huitema A.D., Richel D.J., Dittrich C., Pavlidis N., Briasoulis E., Vermorken J.B., Strocchi E., Martoni A., Sorio R. (2007). Population pharmacokinetics and pharmacodynamics of doxorubicin and cyclophosphamide in breast cancer patients: A study by the EORTC-PAMM-NDDG. Clin. Pharmacokinet..

[39] Ren S., Kalhorn T.F., Slattery J.T. (1999). Inhibition of human aldehyde dehydrogenase 1 by the 4-hydroxycyclophosphamide degradation product acrolein. Drug Metab. Dispos..

[40] Takanashi S., Bachur N.R. (1976). Adriamycin metabolism in man. Evidence from urinary metabolites. Drug Metab. Dispos..

[41] Stewart D.J., Grewaal D., Green R.M., Mikhael N., Goel R., Montpetit V.A., Redmond M.D. (1993). Concentrations of doxorubicin and its metabolites in human autopsy heart and other tissues. Anticancer Res..

[42] Murayama Y., Nagashima M. (1984). [Systemic chemotherapy of mammary carcinoma: Plasma and tissue concentrations of 5-fluorouracil and adriamycin, and result of CAF and CMF therapy of breast cancer. Gan To Kagaku Ryoho.

[43] Danesi R., Fogli S., Gennari A., Conte P., Del Tacca M. (2002). Pharmacokinetic-pharmacodynamic relationships of the anthracycline anticancer drugs. Clin. Pharmacokinet..

[44] Blum R.H., Carter S.K. (1974). Adriamycin. A new anticancer drug with significant clinical activity. Ann. Intern. Med..

[45] Sladek N.E. (1988). Metabolism of oxazaphosphorines. Pharmacol. Ther..

[46] de Jonge M.E., Huitema A.D., Rodenhuis S., Beijnen J.H. (2005). Clinical pharmacokinetics of cyclophosphamide. Clin. Pharmacokinet..

[47] Afsar N.A., Ufer M., Haenisch S., Remmler C., Mateen A., Usman A., Ahmed K.Z., Ahmad H.R., Cascorbi I. (2012). Relationship of drug metabolizing enzyme genotype to plasma levels as well as myelotoxicity of cyclophosphamide in breast cancer patients. Eur. J. Clin. Pharmacol..

[48] Dorr R.T., Lagel K. (1994). Effect of sulfhydryl compounds and glutathione depletion on rat heart myocyte toxicity induced by 4-hydroperoxycyclophosphamide and acrolein in vitro. Chem. Biol. Interact..

[49] Kurauchi K., Nishikawa T., Miyahara E., Okamoto Y., Kawano Y. (2017). Role of metabolites of cyclophosphamide in cardiotoxicity. BMC Res. Notes.

[50] Boyd V.L., Robbins J.D., Egan W., Ludeman S.M. (1986). ^31^P nuclear magnetic resonance spectroscopic observation of the intracellular transformations of oncostatic cyclophosphamide metabolites. J. Med. Chem..

[51] Wang L., Sun Y., Asahi M., Otsu K. (2011). Acrolein, an environmental toxin, induces cardiomyocyte apoptosis via elevated intracellular calcium and free radicals. Cell Biochem. Biophys..

[52] Ismahil M.A., Hamid T., Haberzettl P., Gu Y., Chandrasekar B., Srivastava S., Bhatnagar A., Prabhu S.D. (2011). Chronic oral exposure to the aldehyde pollutant acrolein induces dilated cardiomyopathy. Am. J. Physiol. Heart Circ. Physiol..

[53] McDonald G.B., Slattery J.T., Bouvier M.E., Ren S., Batchelder A.L., Kalhorn T.F., Schoch H.G., Anasetti C., Gooley T. (2003). Cyclophosphamide metabolism, liver toxicity, and mortality following hematopoietic stem cell transplantation. Blood.

[54] Wang D., Wang H. (2012). Oxazaphosphorine bioactivation and detoxification the role of xenobiotic receptors. Acta Pharm. Sin. B.

[55] Pereira-Oliveira M., Reis-Mendes A., Carvalho F., Remiao F., Pinto M., Bastos M.L., Costa V.M. (2019). Doxorubicin is key for the cardiotoxicity of fac (5-fluorouracil + adriamycin + cyclophosphamide) combination in H9c2 differentiated cells. Biomolecules.

[56] Nishikawa T., Miyahara E., Kurauchi K., Watanabe E., Ikawa K., Asaba K., Tanabe T., Okamoto Y., Kawano Y. (2015). Mechanisms of fatal cardiotoxicity following high-dose cyclophosphamide therapy and a method for its prevention. PLoS ONE.

[57] Kimes B.W., Brandt B.L. (1976). Properties of a clonal muscle cell line from rat heart. Exp. Cell Res..

[58] Ruiz M., Courilleau D., Jullian J.C., Fortin D., Ventura-Clapier R., Blondeau J.P., Garnier A. (2012). A cardiac-specific robotized cellular assay identified families of human ligands as inducers of PGC-1α expression and mitochondrial biogenesis. PLoS ONE.

[59] Reis-Mendes A., Gomes A.S., Carvalho R.A., Carvalho F., Remiao F., Pinto M., Bastos M.L., Sousa E., Costa V.M. (2017). Naphthoquinoxaline metabolite of mitoxantrone is less cardiotoxic than the parent compound and it can be a more cardiosafe drug in anticancer therapy. Arch. Toxicol..

[60] Menard C., Pupier S., Mornet D., Kitzmann M., Nargeot J., Lory P. (1999). Modulation of l-type calcium channel expression during retinoic acid-induced differentiation of H9c2 cardiac cells. J. Biol. Chem..

[61] Pereira S.L., Ramalho-Santos J., Branco A.F., Sardao V.A., Oliveira P.J., Carvalho R.A. (2011). Metabolic remodeling during H9c2 myoblast differentiation: Relevance for in vitro toxicity studies. Cardiovasc. Toxicol..

[62] Branco A.F., Sampaio S.F., Moreira A.C., Holy J., Wallace K.B., Baldeiras I., Oliveira P.J., Sardao V.A. (2012). Differentiation-dependent doxorubicin toxicity on H9c2 cardiomyoblasts. Cardiovasc. Toxicol..

[63] Sardao V.A., Oliveira P.J., Holy J., Oliveira C.R., Wallace K.B. (2009). Doxorubicin-induced mitochondrial dysfunction is secondary to nuclear p53 activation in H9c2 cardiomyoblasts. Cancer Chemother. Pharmacol..

[64] Sardao V.A., Oliveira P.J., Holy J., Oliveira C.R., Wallace K.B. (2009). Morphological alterations induced by doxorubicin on H9c2 myoblasts: Nuclear, mitochondrial, and cytoskeletal targets. Cell Biol. Toxicol..

[65] Branco A.F., Pereira S.P., Gonzalez S., Gusev O., Rizvanov A.A., Oliveira P.J. (2015). Gene expression profiling of H9c2 myoblast differentiation towards a cardiac-like phenotype. PLoS ONE.

[66] Repetto G., del Peso A., Zurita J.L. (2008). Neutral red uptake assay for the estimation of cell viability/cytotoxicity. Nat. Protoc..

[67] Almeida D., Pinho R., Correia V., Soares J., Bastos M.L., Carvalho F., Capela J.P., Costa V.M. (2018). Mitoxantrone is more toxic than doxorubicin in SH-SY5Y human cells: A ‘chemobrain’ in vitro study. Pharmaceuticals.

[68] Reers M., Smith T.W., Chen L.B. (1991). J-aggregate formation of a carbocyanine as a quantitative fluorescent indicator of membrane potential. Biochemistry.

[69] Cossarizza A., Baccarani-Contri M., Kalashnikova G., Franceschi C. (1993). A new method for the cytofluorimetric analysis of mitochondrial membrane potential using the J-aggregate forming lipophilic cation 5,5′,6,6′-tetrachloro-1,1′,3,3′-tetraethylbenzimidazolcarbocyanine iodide (JC-1). Biochem. Biophys. Res. Commun..

[70] Franck C., Malfertheiner P., Venerito M. (2017). Safe administration of S-1 after 5-fluorouracil-induced cardiotoxicity in a patient with colorectal cancer. BMJ Case Rep..

[71] Matsubara I., Kamiya J., Imai S. (1980). Cardiotoxic effects of 5-fluorouracil in the guinea pig. Jpn. J. Pharmacol..

[72] Fischel J.L., Formento P., Ciccolini J., Etienne-Grimaldi M.C., Milano G. (2004). Lack of contribution of dihydrofluorouracil and α-fluoro-β-alanine to the cytotoxicity of 5′-deoxy-5-fluorouridine on human keratinocytes. Anticancer Drugs.

[73] Bains O.S., Szeitz A., Lubieniecka J.M., Cragg G.E., Grigliatti T.A., Riggs K.W., Reid R.E. (2013). A correlation between cytotoxicity and reductase-mediated metabolism in cell lines treated with doxorubicin and daunorubicin. J. Pharmacol. Exp. Ther..

[74] Licata S., Saponiero A., Mordente A., Minotti G. (2000). Doxorubicin metabolism and toxicity in human myocardium: Role of cytoplasmic deglycosidation and carbonyl reduction. Chem. Res. Toxicol..

[75] Minotti G., Cavaliere A.F., Mordente A., Rossi M., Schiavello R., Zamparelli R., Possati G. (1995). Secondary alcohol metabolites mediate iron delocalization in cytosolic fractions of myocardial biopsies exposed to anticancer anthracyclines. Novel linkage between anthracycline metabolism and iron-induced cardiotoxicity. J. Clin. Investig..

[76] Minotti G., Recalcati S., Mordente A., Liberi G., Calafiore A.M., Mancuso C., Preziosi P., Cairo G. (1998). The secondary alcohol metabolite of doxorubicin irreversibly inactivates aconitase/iron regulatory protein-1 in cytosolic fractions from human myocardium. FASEB J..

[77] Olson L.E., Bedja D., Alvey S.J., Cardounel A.J., Gabrielson K.L., Reeves R.H. (2003). Protection from doxorubicin-induced cardiac toxicity in mice with a null allele of carbonyl reductase 1. Cancer Res..

[78] Forrest G.L., Gonzalez B., Tseng W., Li X., Mann J. (2000). Human carbonyl reductase overexpression in the heart advances the development of doxorubicin-induced cardiotoxicity in transgenic mice. Cancer Res..

[79] Minotti G., Cairo G., Monti E. (1999). Role of iron in anthracycline cardiotoxicity: New tunes for an old song?. FASEB J..

[80] Xie Y., Hou W., Song X., Yu Y., Huang J., Sun X., Kang R., Tang D. (2016). Ferroptosis: Process and function. Cell Death Differ..

[81] Zhou F., Hao G., Zhang J., Zheng Y., Wu X., Hao K., Niu F., Luo D., Sun Y., Wu L. (2015). Protective effect of 23-hydroxybetulinic acid on doxorubicin-induced cardiotoxicity: A correlation with the inhibition of carbonyl reductase-mediated metabolism. Br. J. Pharmacol..

[82] Sun Y., Ito S., Nishio N., Tanaka Y., Chen N., Isobe K. (2014). Acrolein induced both pulmonary inflammation and the death of lung epithelial cells. Toxicol. Lett..

[83] Horton N.D., Biswal S.S., Corrigan L.L., Bratta J., Kehrer J.P. (1999). Acrolein causes inhibitor κB-independent decreases in nuclear factor κB activation in human lung adenocarcinoma (A549) cells. J. Biol. Chem..

[84] Biswal S., Acquaah-Mensah G., Datta K., Wu X., Kehrer J.P. (2002). Inhibition of cell proliferation and AP-1 activity by acrolein in human A549 lung adenocarcinoma cells due to thiol imbalance and covalent modifications. Chem. Res. Toxicol..

[85] LoPachin R.M., Gavin T., Petersen D.R., Barber D.S. (2009). Molecular mechanisms of 4-hydroxy-2-nonenal and acrolein toxicity: Nucleophilic targets and adduct formation. Chem. Res. Toxicol..

[86] Damiani R.M., Moura D.J., Viau C.M., Caceres R.A., Henriques J.A., Saffi J. (2016). Pathways of cardiac toxicity: Comparison between chemotherapeutic drugs doxorubicin and mitoxantrone. Arch. Toxicol..

[87] Hansen J.M., Go Y.M., Jones D.P. (2006). Nuclear and mitochondrial compartmentation of oxidative stress and redox signaling. Annu. Rev. Pharmacol. Toxicol..

[88] Gervasi P.G., Agrillo M.R., Citti L., Danesi R., Del Tacca M. (1986). Superoxide anion production by adriamycinol from cardiac sarcosomes and by mitochondrial nadh dehydrogenase. Anticancer Res..

[89] Olson R.D., Mushlin P.S., Brenner D.E., Fleischer S., Cusack B.J., Chang B.K., Boucek R.J. (1988). Doxorubicin cardiotoxicity may be caused by its metabolite, doxorubicinol. Proc. Natl. Acad. Sci. USA.

[90] Boucek R.J., Olson R.D., Brenner D.E., Ogunbunmi E.M., Inui M., Fleischer S. (1987). The major metabolite of doxorubicin is a potent inhibitor of membrane-associated ion pumps. A correlative study of cardiac muscle with isolated membrane fractions. J. Biol. Chem..

[91] Zhang D., Ma J. (2018). Mitochondrial dynamics in rat heart induced by 5-fluorouracil. Med. Sci. Monit..

